# Diminished Auditory Responses during NREM Sleep Correlate with the Hierarchy of Language Processing

**DOI:** 10.1371/journal.pone.0157143

**Published:** 2016-06-16

**Authors:** Meytal Wilf, Michal Ramot, Edna Furman-Haran, Anat Arzi, Yechiel Levkovitz, Rafael Malach

**Affiliations:** 1 Department of Neurobiology, Weizmann Institute of Science, Rehreovot 76100, Israel; 2 Department of Biological Services, Weizmann Institute of Science, Rehovot 76100, Israel; 3 Shalvata Mental Health Care Center, School of Medicine, Tel Aviv University, Tel Aviv, Israel; University of Oxford, UNITED KINGDOM

## Abstract

Natural sleep provides a powerful model system for studying the neuronal correlates of awareness and state changes in the human brain. To quantitatively map the nature of sleep-induced modulations in sensory responses we presented participants with auditory stimuli possessing different levels of linguistic complexity. Ten participants were scanned using functional magnetic resonance imaging (fMRI) during the waking state and after falling asleep. Sleep staging was based on heart rate measures validated independently on 20 participants using concurrent EEG and heart rate measurements and the results were confirmed using permutation analysis. Participants were exposed to three types of auditory stimuli: scrambled sounds, meaningless word sentences and comprehensible sentences. During non-rapid eye movement (NREM) sleep, we found diminishing brain activation along the hierarchy of language processing, more pronounced in higher processing regions. Specifically, the auditory thalamus showed similar activation levels during sleep and waking states, primary auditory cortex remained activated but showed a significant reduction in auditory responses during sleep, and the high order language-related representation in inferior frontal gyrus (IFG) cortex showed a complete abolishment of responses during NREM sleep. In addition to an overall activation decrease in language processing regions in superior temporal gyrus and IFG, those areas manifested a loss of semantic selectivity during NREM sleep. Our results suggest that the decreased awareness to linguistic auditory stimuli during NREM sleep is linked to diminished activity in high order processing stations.

## Introduction

The search for neuronal correlates of sensory percepts typically examines changes in awareness to different categories of sensory stimuli (including meaningless or noisy stimuli) while subjects are awake. However, these types of studies do not address the question of how the responses to these stimuli change as participants move from a conscious to non-conscious states. To that end the phenomenon of naturally occurring sleep provides a powerful and potentially informative experimental system [1]. Sleep, and specifically the slow-wave, non- rapid eye movement stage (NREM), is linked to a massive disruption in conscious awareness [2]. Thus, comparing sensory brain responses during the awake state and sleep could point to the neuronal correlates of sensory perception and awareness in the human brain.

Indeed a number of studies have addressed the issue of sensory processing during sleep. Both animal electrophysiology research and human event-related potential (ERP) studies have found little change in prethalamic transmission of auditory signals [3–5]. Conversely, at the cortical level, a significant sleep-induced modulation of signal properties such as latency and amplitude of the ERP signal, or firing rates of single neurons has been documented in some studies [4, 6–8], while a recent animal study has found preserved auditory response in primary auditory cortex [9]. Positron emission tomography (PET) studies revealed a general decrease in regional cerebral blood flow during deep NREM sleep compared to wakefulness [10]. Using ERPs some evidence for semantic-related effects and auditory deviance detection have been reported during sleep, though fine differences between semantic levels were lost [11, 12]. Additionally, Perrin et al. [13] found that hearing one's own name elicits an enhanced ERP response compared to other proper names during rapid eye movement (REM) and light NREM sleep. However, the role of one's own name as a semantic object is not well established [14].

A recent study by Strauss et al. [15] examined the effect of sleep on the processing of global and local feature changes in auditory stimuli. They found that during sleep the magnetoencephalographic (MEG) response to local changes in auditory features (a single vowel change) remains evident while the response to changes in the global regularities of the stimulus (a change of vowel sequence) vanishes.

With the advent of brain imaging with functional magnetic resonance (fMRI) it became possible to map more precisely the anatomical layout and functional selectivity of the sleep-induced modulations in sensory processing. Indeed, several studies combining electroencephalography (EEG) and fMRI found reduced auditory activation and even deactivation in cortical areas in response to acoustic stimulation during NREM sleep [16–18]. This deactivation was correlated with the existence of EEG events thought to be a mechanism of sleep protection (k-complexes) [16], leading the authors to suggest that it also serves as a sleep protective mechanism. However, the sleeping brain still exhibits selectivity to rare tones over frequent tones during NREM sleep, but this selectivity is expressed as deactivation of motor areas, rather than as increased auditory activation which is the typical cortical response during the waking state [17]. Other studies using low-level auditory stimuli have shown that the amount of auditory blood oxygenation level dependent (BOLD) activation in response to simple tones during NREM sleep is correlated with the existence of spindles or the phase of K-complexes in the EEG trace [19, 20]. Probing for a change in global brain dynamics, functional connectivity analyses of BOLD-fMRI revealed a decrease in connectivity between posterior and anterior regions, and increased local clustering during sleep [21–23]. However, we have previously demonstrated that the functional organization, as reflected in coherent patterns of activations, seen during wakefulness in sensory cortices is remarkably preserved during sleep, especially during the NREM phases [24]. Strong connectivity between the two hemispheres of auditory cortex was also found during sleep. These interhemispheric correlations actually increased during REM and stage 2 sleep [25].

Of particular relevance to the present work is the study by Portas et al. [26], who demonstrated, on the one hand, insensitivity to sleep-awake transitions in primary auditory cortex, and on the other hand, substantial modulation in frontal cortex regions. These results suggest an axis of increased sleep modulation along the cortical hierarchy of processing. However, Portas et al. used both linguistic (own name) and non-linguistic (beep) items—that were too diverse to allow a systematic mapping along the hierarchy of linguistic processing.

In the present work we used sentences of increasing linguistic complexity during both wakefulness and sleep to map the linguistic chain of processing from auditory thalamus to inferior frontal gyrus (IFG; Broca's area) in the frontal lobe. Our results show that the impact of sleep on auditory activation increased from thalamic, through early auditory stations, to high order linguistic processing areas in the frontal cortex, where activity appeared to be completely abolished. Thus, our results are compatible with the hypothesis that the main sleep-induced gating process lies at the transition from low level to high order linguistic processing centers in the human brain. These results provide further support to the critical role of high order cortical processing in perceptual awareness.

## Results

The present study was based on ten participants who were scanned in a 3T MR scanner during states of wakefulness and sleep. Table 1 summarizes the statistics of the scans for each participant. After excluding scans with movement artifacts or ambiguous sleep staging (see methods) we obtained 634 minutes of BOLD imaging (63 ± 24 min per participant) that were collected during the auditory sequence in the night sessions. Out of which 377 minutes were collected during wakeful periods and 257 minutes during NREM sleep periods (again, excluding scans with equivocal arousal state). The awake and sleep periods alternated during the night in each participant, and there was no clear state order effect (32 of the times the awake period occurred after the sleep period, while 34 of the times the opposite occurred). However, 9 out of 10 participants were awake during the first presentation of the auditory sequence. Sleep staging was based on pulse/heart rate monitoring, in conjunction with eye tracking and EMG recordings. S1 Fig shows a comparison of this method with more conventional EEG recordings.

**Table 1:** Summary of fMRI scan duration for each participant during sleep and wakefulness.

Participant ID	All awake scan time (min)*	All sleep scan time (min)*	Raw auditory awake (min)	Raw auditory sleep (min)	Clean auditory awake (min)**	Clean auditory sleep (min)**
**s01**	81	126	27	36	26	36
**s02**	234	63	72	9	63	9
**s03**	144	63	45	9	36	8
**s04**	63	135	18	36	14	33
**s05**	99	126	18	18	12	5
**s06**	243	36	63	9	53	9
**s07**	144	99	45	36	44	34
**s08**	126	117	54	36	51	35
**s09**	99	162	36	36	26	33
**s10**	171	153	54	54	52	54
**Total**	**1404**	**1080**	**432**	**279**	**377**	**256**

* Sum of 9 min sequences that obtained unequivocal staging of either awake or sleep

** **C**lean = after excluding segments with head motion

Since no scan showed the presence of REMs or low voltage EMG accompanied by increased heart rate, no scan was classified as REM sleep. Although our sessions were conducted in the second half of the night, where REM sleep is normally more dominant, the absence of clear REM sleep could be likely attributed to the uncomfortable conditions in the scanner. Note that our sleep staging method could only provide a rough subdivision into NREM sleep/REM sleep/wakefulness. S1 Table shows the confusion matrix of our scoring method, compared with traditional EEG sleep scoring, from the same validation data set presented in S1 Fig (see methods for details of validation analysis). According to participants’ self-reports and the scoring results, participants' sleep was fragmented; there were many disruptions of sleep continuity followed by long periods of wakefulness. Most (26/42) sleep epochs were under 30 minutes long, and only 4/42 sleep epochs were over 50 minutes long, far less than is usually observed in sleep studies in a more sleep conducive environment. S2 Fig shows a summary of sleep epoch length for all participants, aggregated across nights.

**Fig 1:**
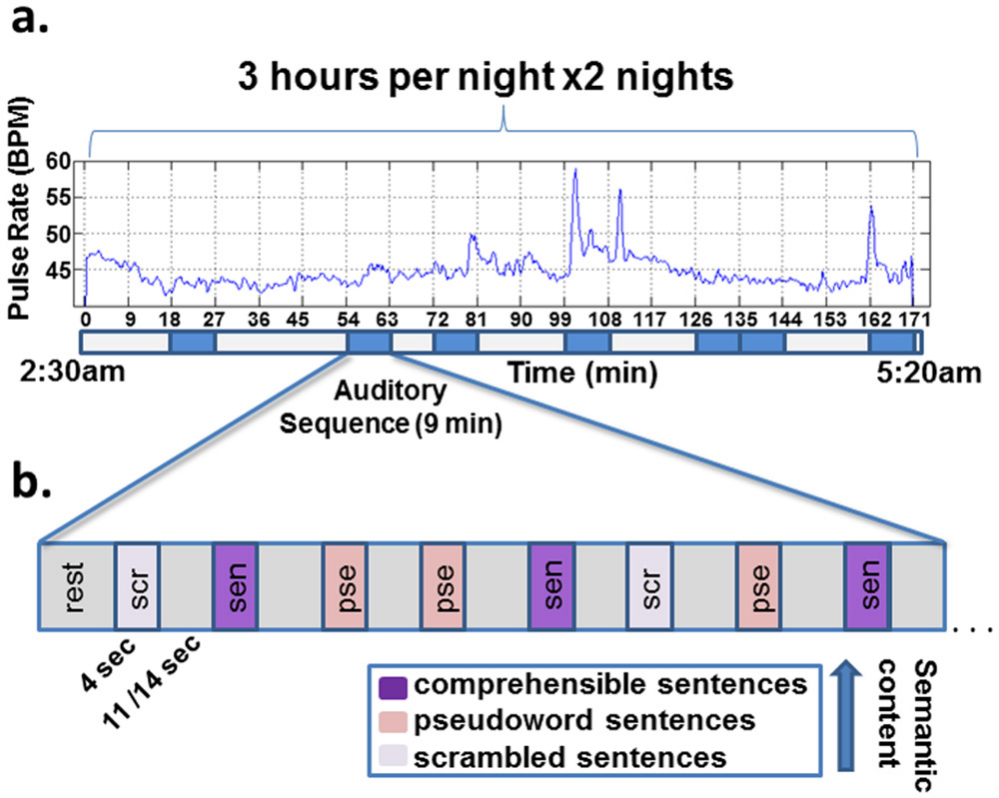
Schematic illustration of the experimental procedure. The figure depicts an example of a night session. *(a)* Pulse rate recorded during the fMRI session. Auditory sequence periods, denoted by blue rectangles below the x axis, were sparsely presented during the night. *(b)* An example of a single auditory sequence. Auditory stimuli alternated with periods of silence, which served as baseline. Legend describes the types of auditory stimuli used (see text for details).

Fig 1 depicts the structure of the auditory stimuli and the type of auditory material that was presented (see methods for details). Participants were exposed to three main categories of auditory stimuli, aimed at emphasizing different levels of linguistic processing: comprehensible sentences, pseuodoword sentences, and scrambled sentences (see methods).

**Fig 2:**
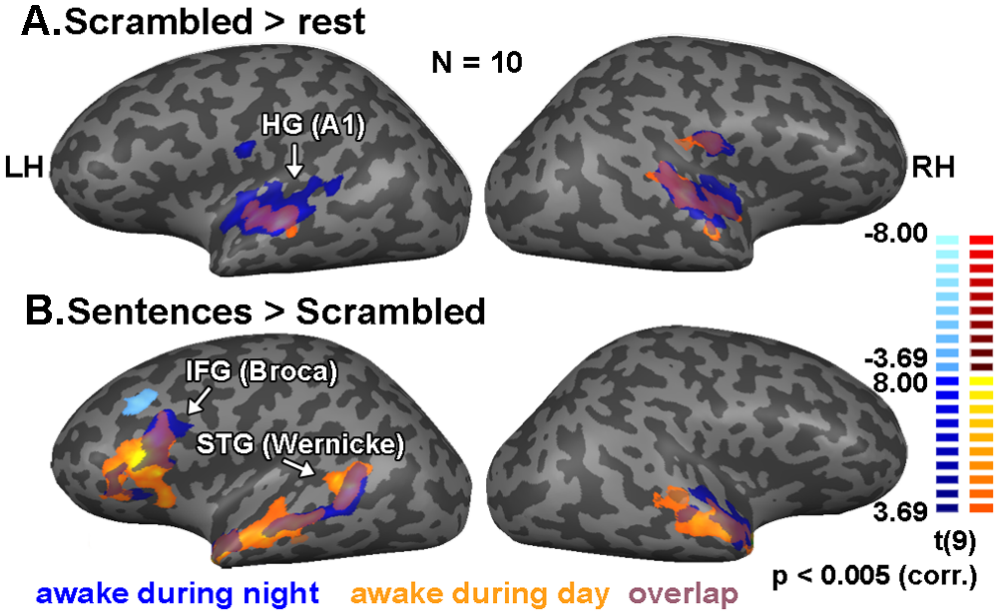
Cortical responses to auditory stimuli during wakefulness. Statistical parametric maps of GLM multi participant (n = 10) random effect analysis. Color coding denotes t values. *(a)* Response to scrambled sentences versus rest during wakefulness in the night session (blue shades), and in auditory localizer scans (orange shades). Note the high proportion of overlap (purple shades) *(b)* Regions showing preferred activation for comprehensible sentences over scrambled sentences in awake periods during the night session and in auditory localizer scans. HG = Heschl’s gyrus, IFG = inferior frontal gyrus, STG = superior temporal gyrus. Color bars denote the maps t values during the night (left scale) and daytime localizer (right scale) sessions.

**Fig 3:**
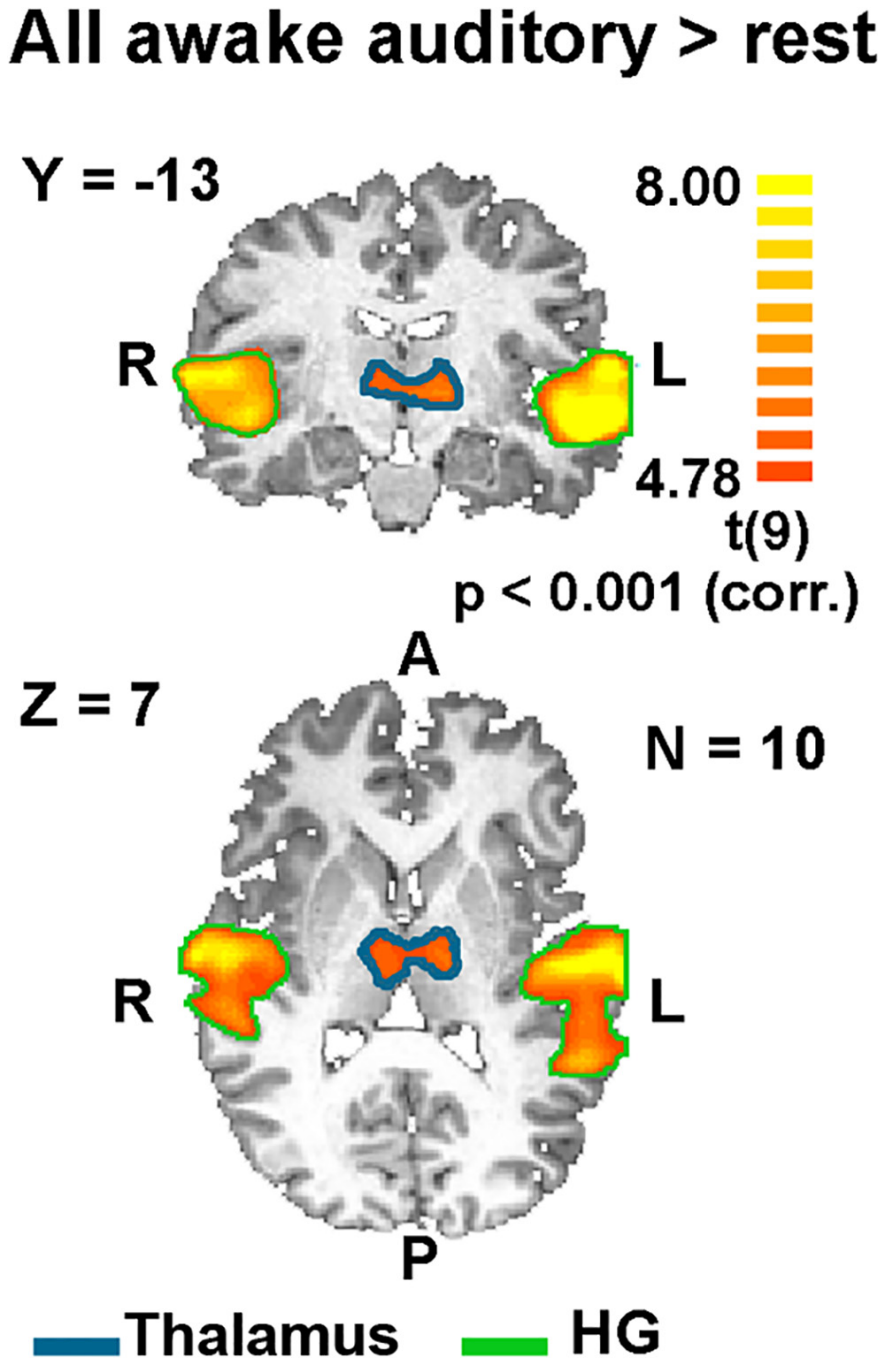
Subcortical responses to all auditory stimuli versus rest during wakefulness (both awake during the night and the localizer session). Statistical parametric maps of GLM multi participant (n = 10) random effect analysis. Color coding denotes t values. The map shows response to all three types of auditory stimuli during all wakefulness segments in coronal (top) and axial (bottom) slices. HG = Heschl’s gyrus.

As expected from previous studies of auditory language processing, our stimuli were highly effective in activating the expected auditory and language-related areas during the awake state (Fig 2 and Table 2). Fig 2 depicts the activation patterns, during the awake state, associated with early and high order auditory and language processing, respectively. We observed robust activation in bilateral superior temporal cortex, including Heschl's gyrus (HG; BA 41, 42) corresponding to primary and secondary auditory cortices in response to each of the stimulus categories, including the scrambled sentences (low-level auditory stimulus; see Fig 2*a*). To detect language processing-related regions, we contrasted the BOLD response to comprehensible versus scrambled sentences. This comparison revealed activations in superior temporal gyrus (STG; BA 22), more lateralized to the left hemisphere, corresponding to Wernicke's area, and in left inferior frontal gyrus (IFG; BA 44), corresponding to Broca's area (Fig 2*b* and Table 2). As can be seen, the activation pattern during wakeful periods during the night extensively overlapped that of the awake session during the day (Fig 2). However, there was a small decrease in activation spread (as evident in Fig 2*b*), that might have resulted from drowsiness or adaptation due to repetitions during the night. In addition, we noted significant auditory activation during the awake state in the thalamus (see Fig 3).

**Table 2:** Regions activated by the task during wakefulness and sleep.

Anatomical location	Left Hemisphere				Right Hemisphere			
	X	Y	Z	*t*-value	X	Y	Z	*t*-value
**Sen + pse + scr > rest awake**								
Thalamus	-10	-11	3	5.2	7	-14	6	5.2
Heschl's gyrus	-59	-15	4	10.4	52	-14	9	7.9
**Sen > scr awake**								
Superior temporal gyrus	-62	-44	2	10.4	56	-1	-5	6.4
Inferior frontal gyrus	-46	17	22	7.9	38	18	24	4.4
**Sen + pse + scr > rest sleep**								
Thalamus	-5	-19	9	5	9	-21	12	5
Heschl's gyrus	-46	-19	-2	4.5	NS	NS	NS	NS
**Sen > scr sleep**								
Superior temporal gyrus	NS	NS	NS	NS	61	-17	3	4

Coordinates are reported in Talairach space, and represent the location of the peak of activation. The t-values are reported for the most activated voxels. Only significantly activated regions (p < 0.005, Monte-Carlo corrected) are presented. NS = non-significant, sen = comprehensible sentences, pse = pseudoword, scr = scrambled

Using stimulus specific activations during the day as an independent localizer, we defined, in each participant individually, four regions of interest (ROIs) as follows: the thalamus ROI was chosen as significantly activated voxels for all the auditory stimuli versus rest within the anatomical location of the thalamus; Primary auditory cortex was defined as the significantly activated voxels in scrambled sentences versus rest contrast in the temporal lobe; Wernicke’s and Broca’s areas were selected using a contrast of comprehensible sentences versus scrambled sentences in the relevant anatomical locations (STG and IFG respectively).

**Fig 4:**
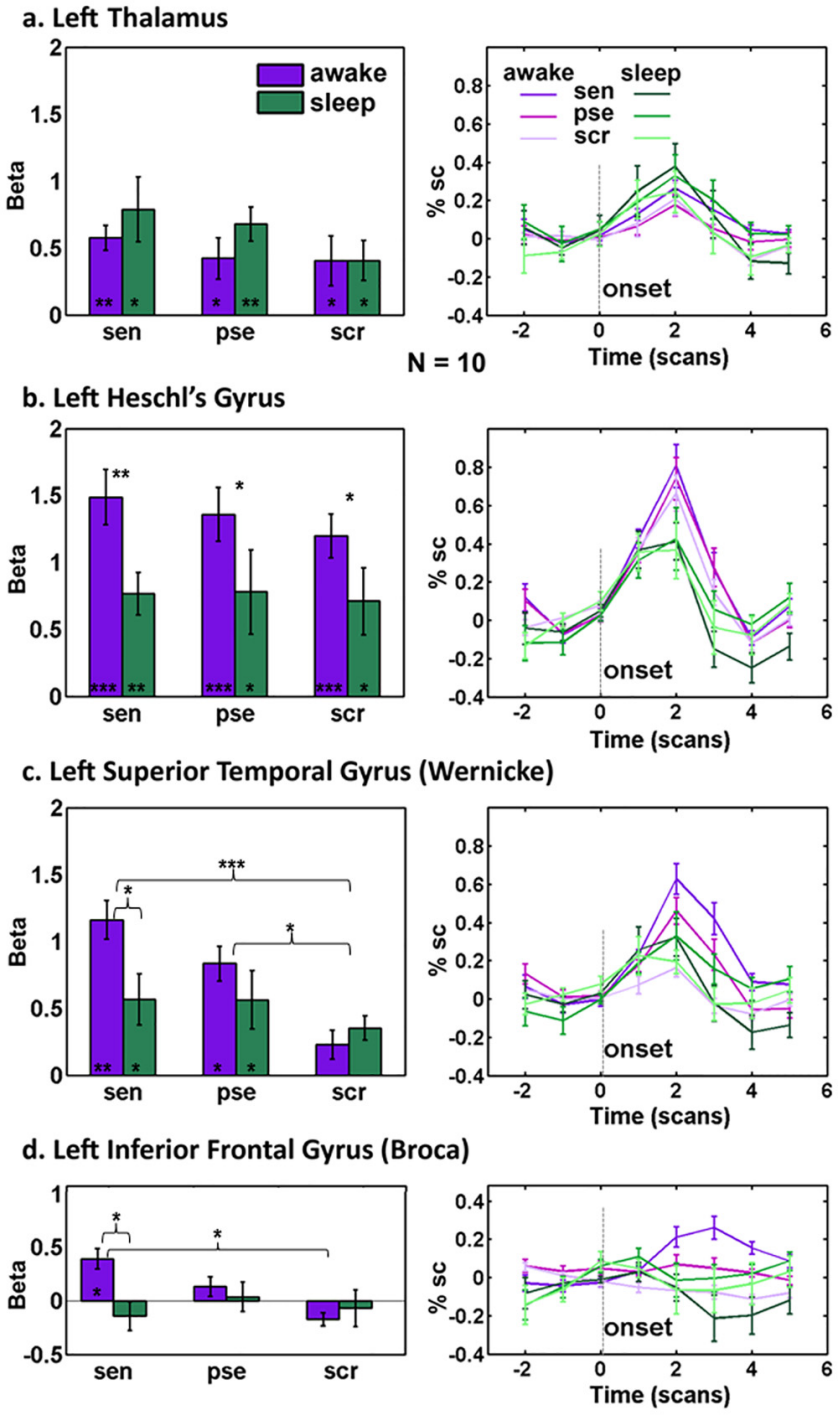
ROI analysis of all three types of auditory stimuli in wakefulness and sleep during night sessions. *(a-d left)* Mean beta values (n = 10) after GLM analysis for each stimulus category in wakefulness (purple) and sleep (green) in *(a)* thalamus *(b)* left Heschl’s gyrus (primary auditory cortices) *(c)* left superior-temporal gyrus (Wernicke), *(d*, *left 3 bars)* left inferior frontal gyrus (Broca), and *(d*, *right bar)* comprehensible sentences category for only low activated voxels in HG. *(a-d right)* Averaged hemodynamic response curves of percent signal change for the auditory stimuli in sleep and wakefulness across participants (awake: purple shades, sleep: green shades; see legend). Errorbars represent standard error of mean (SEM). Dashed lines denote the onset of a stimulus event. sen = comprehensible sentences, pse = pseudoword, scr = scrambled. Significance values of Tukey tests or one-sampled t-tests (corrected) are shown by asterisks, above the bars to denote differences between categories / states, and at the bottom of the bars to denote significance above baseline: * p < 0.05 ** p < 0.005 *** p < 0.0005.

**Fig 5:**
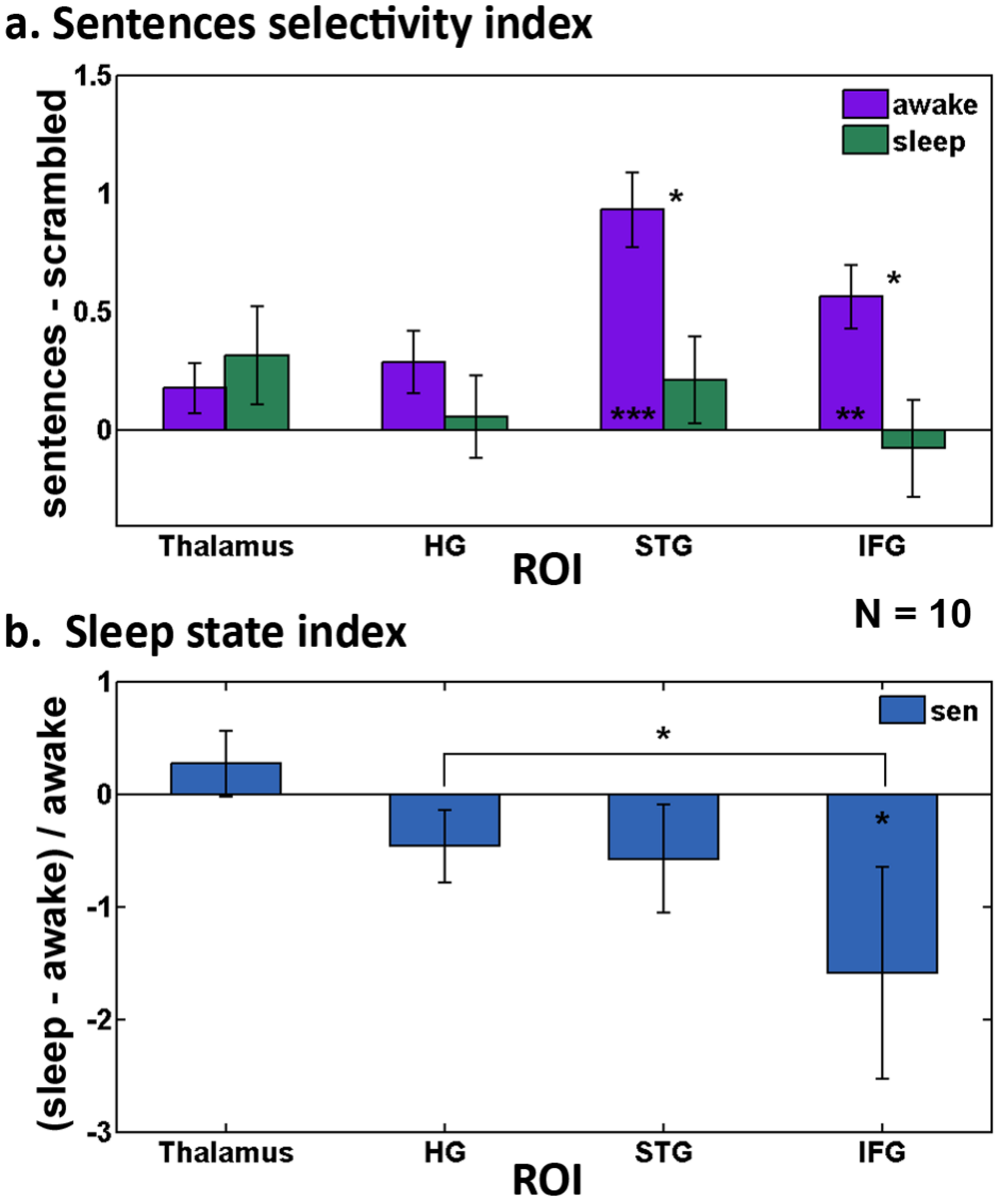
Indices quantifying the effect of sleep on the different ROIs. Beta values were used to calculate the indices (n = 10). *(a)* Mean index assessing semantic selectivity during wakefulness (purple) and sleep (green), calculated by subtracting betas of scrambled from comprehensible sentences in each of the ROIs. *(b)* Median index measuring the effect of sleep on the response to comprehensible sentences, calculated by subtracting awake betas from sleep betas and dividing by awake betas for the comprehensible sentences category. Note the graded decrease in the index values moving along the hierarchy of semantic processing ROIs. sen = comprehensible sentences. Statistical specifications are the same as in Fig 4.

Fig 4 depicts the auditory activations in the four ROIs during the night in both the awake and sleep states for the left hemisphere (a similar analysis for the right hemisphere is presented in S3 Fig, a summary of the activated regions can also be found in Table 2). Note that for all multi-subject ROI analyses we first calculated two single mean beta values for each participant, one for sleep and the other for wake (see methods). As can be seen, the cortical auditory responses tended to be reduced during sleep, and the effect appeared to be accentuated in high order processing stages. More specifically, the thalamic activation showed no significant difference in activation level between sleep and wakefulness (Fig 4*a*; F(1,9) = 0.9, p = 0.4), but when moving downstream in the auditory pathway to the primary auditory cortex, a significant decrease in BOLD signal was observed in all the auditory categories (F(1,9) = 6.3, p = 0.03), with no difference between the categories (Fig 4*b*; F(2,9) = 1.4, p = 0.3). Although decreased, sleep activations were nevertheless significantly higher than baseline for all categories (p < 0.05 Bonferroni corrected, one-sampled t-test), indicating a residual auditory response during sleep. Language processing regions in STG showed a significant reduction in activation to comprehensible sentences during sleep, and no distinction between the different categories (Figs 4*c* and 5*a*; F(2,9) = 4.4, p = 0.03, interaction between state and category). This was in contrast with the graded activation according to the level of semantic content found in this region in the awake state (Fig 4*c*; F(2,9) = 13.2, p = 0.0003, category main effect). In IFG the response to comprehensible sentences was completely abolished (Fig 4*d*; p > 0.09), contrary to the graded activation according to semantic level during wakefulness (F(2,9) = 4.4, p = 0.03, category main effect; F(2,9) = 5.7, p = 0.01, interaction), suggesting a more dramatic influence of sleep on this frontal ROI.

Notably, since in most cases the participants were awake during the first presentation of the auditory sequence (9 out of 10), it could be argued that the diminished response in primary auditory cortex during sleep compared to wakefulness was the result of adaptation. To rule out this possibility, we performed the same analysis while omitting the first wakefulness run of each participant, and still found a significant decrease in primary auditory cortex activation during sleep (F(1,9) = 5.1, p = 0.05, state factor in repeated-measures ANOVA). To further directly test the effect of presentation order, we calculated the correlation between the presentation order and the beta values in HG (for the first and second night separately), pooling together all participants. This analysis showed no correlation between betas and presentation order (r = -0.1, p = 0.53 on night 1; r = 0.06, p = 0.71 on night 2).

Furthermore, to control for the possibility of incorrect labeling of the sequences as sleep or wakefulness, we performed a permutation test comprised of shuffling the sleep-wake labels within each participant (see methods). We then compared the ANOVA statistic for state factor in primary auditory cortex for each of these random permutations with the ANOVA statistic generated by the original labels (F(1,9) = 6.3). The results of this test showed that our original labeling based on the sleep-wake indicator was significantly better than the randomly shuffled labels (p = 0.026; see methods). We repeated the same analysis for our two other ROIs, which showed a similar trend that did not quite reach significance (Wernicke’s region p = 0.13, Broca’s region p = 0.055).

Since sleep staging in this study was based on cardiac activity as measured by changes in pulse/heart rate, we conducted a correlation analysis between the beta values and mean heart rate of each run, to test for any trivial link of this variable to our results. This analysis (carried out separately for sleep/wake) revealed no such link (awake state: r = -0.11, p = 0.48. Sleep state: r = 0.06, p = 0.76).

Finally, to quantify the effect of sleep on the activations in the regions along the hierarchy of auditory and linguistic processing, we calculated a sleep index (calculated from beta values as index = (*sleep-awake)/awake*) for the comprehensible sentences category (Fig 5*b*). The index revealed a differential effect of sleep on the response to comprehensible sentences—so that response to comprehensible sentences gradually diminished along the hierarchy of linguistic processing (Fig 5*b*; p < 0.005, repeated-measures ANOVA).

## Discussion

In the present study we have found that being asleep significantly reduced BOLD activations induced by auditory and linguistic stimuli in the human cortex. However, the effect of sleep was differential. By using stimuli that represent various levels of audio/linguistic processing we found that the impact of sleep was more pronounced at higher levels of the cortical hierarchy: auditory responses in the thalamus did not show a significant sleep-induced modulation. By contrast, early auditory areas showed a significant reduction in auditory responses. Finally, language processing regions showed a reduced or completely abolished response to comprehensible stimuli, accompanied by a lack of differentiation between different semantic levels (see Fig 5). Though we expected to see thalamic activations from auditory stimuli primarily in the medial geniculate nucleus (MGN), the thalamic activation we found was more frontal and anterior than traditional atlas-based locations of the MGN. This could potentially be the result of the relatively poor signal quality in the thalamus, compounded by the spatial limitations of accurately localizing a small nucleus with averaged group analysis in standard space. Alternately, other thalamic nuclei such as the medial pulvinar may have also been activated by our task, thus further moving the focus of activation [27].

Although significantly diminished, a residual signal that was not entirely abolished by sleep could nevertheless be observed, particularly in early regions during sleep. This signal may reflect auditory processing that still persisted in these areas despite the lack of auditory awareness [28], or could potentially be a confound of our imperfect staging technique.

Another possible factor that may have contributed to our results could be a partial gating in the transition from the thalamus to the cortex. Such a partial block in the relay of information from thalamus to auditory cortex could account for the decrease in auditory cortex activation, a decrease that was later amplified in the transition to higher language processing regions in the frontal cortex. This hypothesis is compatible with animal studies and with human data showing thalamo-cortical asynchrony in the descent to sleep [4, 22, 29, 30]. However, the loss of language selectivity in STG and the complete abolishment of responses in IFG, suggest an additional effect of sleep on these high-order areas, which could not be explained by mere thalamic gating. Furthermore, recent animal studies question the existence of thalamic gating by showing auditory responses in primary auditory regions during sleep [6, 9].

Additionally, the graded reduction of response to auditory stimuli (from auditory cortex through Wernicke’s in the STG to Broca’s in IFG) could also reflect some changes in connectivity between the regions that might occur during sleep (Fig 4). At this stage, because of the difference in the baseline level of activation of these regions during wake which might lead to a floor effect, we cannot tell whether the decreased activity during sleep is being driven by reduced functionality of the region or by reduced input. However, the lack of differentiation between categories and the loss of selectivity for sentences, even when residual input still exists (like in the case of Wernicke’s region) suggests some changes in functionality do occur (Fig 5*a*). It should be noted though, that despite the loss of selectivity in Wernicke’s, only Broca’s area showed a significant sleep state index (Fig 5*b*). Furthermore, surprisingly, activations in the thalamus were not only preserved, but also somewhat enhanced during sleep (Fig 4 and S3 Fig). Significant enhancement was shown only for the right thalamus, in the pseudo sentences condition, but the same trend was evident for both thalamic ROIs, in both the sentences and pseudo sentences conditions. It might therefore be hypothesized from these results that some bottom up processing is preserved during NREM sleep (explaining the residual activations for all kinds of auditory stimuli in Wernicke’s), but top-down processes are eliminated, explaining both the lack of selectivity for sentences in Wernicke’s, and the lack of initiation of a motor response in Broca’s, which is most likely the result of a top-down process. This theory is in line with previous research showing loss of top-down processes in sleep [31].

In the current study indicating wakefulness or sleep relied on heart rate monitoring combined with eye tracking and EMG rather than the more conventional use of EEG recordings. This approach produced substantially less discomfort, allowing a greater success in sleep induction, however, the validity of this approach for sleep staging is less established compared to more conventional EEG recordings (but see [32]). While it is quite likely that our approach was less accurate than the EEG-based method, it should be noted that we have validated it carefully in 20 additional subjects, and have shown that it was highly accurate as long as the staging was limited to differentiating NREM from awake/REM sleep (see methods, S1 Fig and S1 Table). By combing the pulse-based staging approach with EMG and video monitoring we were successful in further differentiating any possible REM episodes from the wakeful state.

Nevertheless, it could be argued that our results may somehow be the outcome of inappropriate identification of the NREM timing. While it is quite plausible that some errors in timing of the NREM periods may still have occurred in our study it is unlikely that our results can be explained by such improper classification to wakefulness or sleep. As evident from the result of our permutation test, random assignments of sleep/wake labels resulted in a significantly weaker effect than the sleep-related reduction of activity in primary auditory cortex that we found with the original labels, strongly arguing for the effect being sleep-related, and for our labels being overall correct. However, this permutation test only approached significance for the other two ROIs. This might be because of the attenuation of the auditory signal along the processing hierarchy (discussed below), which resulted in overall weaker signals in those areas and therefore a decreased signal to noise ratio, making these regions more sensitive to slight misclassifications of sleep/wake.

Thus, the finding of significantly diminished BOLD responses during sleep cannot be explained by a mis-categorization of sleep events as awake ones and vice versa. The fact that despite such potential "blurring" of the differences we found a significant and selective reduction in BOLD activations indicates that the effect was robust enough to overcome the added noise imposed by mis-categorization of wakeful events as sleep ones. Furthermore, if indeed there was a substantial mixing of the staging periods, this suggests that the observed reduction in BOLD activation may have underestimated the true sleep-related reduction.

As discussed above, our classification method was nevertheless able to reliably differentiate experimental segments performed during NREM sleep from those during wakefulness or REM sleep, as evident in the test data (S1 Fig). However, our pulse data was not sensitive to transient events such as spindles or k-complexes. Thus, the issue of the well-known effects of these events on the processing of incoming stimuli [19, 33] was not examined in the present study. Our staging method could also not disassociate the different sleep stages, and in particular SWS from stage 1 or 2 sleep, which some research suggests might preserve different levels of semantic processing [11, 34, 35] Further research is needed to establish the relation of the various stages and transient events during sleep to semantic processing.

Our strategy for differentiating REM sleep and wakefulness (both characterized by high heart rate) was based on assessing EMG and eye movement data. The fact that we did not observe any REM period during our auditory experiments might seem surprising, but it should be noted that the MRI scanner is a drastically different sleep environment compared to natural conditions, both in terms of noise and confinement. Studies of nocturnal sleep under noisy conditions (such as intensive care units) have found a disruption of sleep continuity and an alteration of sleep stage pattern [36, 37]. Freedman et al. [36] reported a total absence of REM sleep for most patients recorded (12 out of 17) and a significant reduction of REM quantity for the remaining patients. It is therefore not surprising that under our experimental setup participants tended to wake up more often and did not exhibit prolonged REM activity. Furthermore, the fMRI activity pattern during the wakefulness segments was largely similar to that evident in our localizer experiment (Fig 2) and to that reported in previous studies (Rodd et al 2005, Davis et al. 2007). The fMRI activity during the sleep segments, however, was decreased even when compared to other wakefulness data.

An additional methodological concern deriving from our staging method might be that the change in heart rate itself was the cause for the reported effect. However, as stated in the results, a direct comparison between heart rate and beta values showed no correlation. This is not surprising, as studies have shown that the effects of changes in heart rate on BOLD signal are prominent in regions related to autonomic control, such as anterior cingulate cortex and amygdale, and around large vessels [38–41], and therefore less relevant to the ROIs analyzed in this study. Moreover, heart rate fluctuations account for only a small percent of the variability of the BOLD signal in both resting activity and task [39, 42]. Finally, the main modulation found in our study was quite localized, arguing against a more global pulse-rate related effect.

Because the same stimuli were repeated throughout the night, it might be argued that the decrease in the BOLD signal during sleep was due to repetition suppression—i.e. fMRI adaptation following multiple repetitions [43]. Indeed, we saw some reduction in the activation for the awake runs during the night compared with the auditory localizer runs, which could derive from either drowsiness, some adaptation, or potential mixing of short sleep periods. However, adaptation cannot account for the differences between wakefulness and sleep during the night, as even when we removed the first wakefulness run, thus removing the potential bias towards higher activation during wakefulness, we still found a significant decrease of primary auditory activation during sleep (see results).

Our results are in accordance with recent findings that processing of global features of auditory stimuli (albeit not stimuli requiring semantic processing) diminishes during sleep [15]. This breakdown of global connectivity during sleep has been demonstrated by Massimini and colleagues, in a series of TMS experiments showing that the TMS pulse has a far more local spread during NREM sleep, suggesting that the sleeping brain is composed of causally independent modules [44–46]. The nature of the stimuli we used required some level of global processing; therefore the lack of global feature processing could also affect the ability of the cortex to process stimuli with semantic content. This could also explain the increased attenuation of the auditory signal that we find along the auditory processing hierarchy. The breakdown of effective connectivity could result in the response to the auditory stimuli remaining circumscribed in primary auditory cortex, due to a loss of cortico-cortical connectivity.

A similar link to that reported here, between loss of perceptual awareness and specific reductions in high order language areas was reported during anesthesia. Davis et al. [28] have shown accentuation of the sleep effect along the language hierarchy with increasing levels of sedation. Indeed, our results extend those of Davis et al. by revealing an effect of state change also in Wernicke’s region and auditory cortex, as well as a loss in selectivity to meaningful stimuli. This is in accordance with the notion that during natural sleep the brain is in need of more attenuation of incoming signals in order to protect the sleep state. It is important to emphasize that a substantial physiological difference exists between natural sleep and anesthesia [47]. What is common to natural sleep and anesthesia is the loss of consciousness, so the similarity in the effect under these two conditions is in line with the hypothesis that the BOLD reduction is linked to loss of perceptual awareness in both cases.

Similarly to anesthesia, vegetative state patients showed preserved primary auditory cortex activity and reduced blood flow in high order association regions in response to auditory stimuli [48]. In the study by Portas et al. [26] no effect of sleep was reported in early auditory cortex, but only in frontal regions. By contrast, in a study by Czisch et al. (2002), the authors did find a decrease in auditory activation during sleep. The reason for the discrepancy may be attributed to the different nature of the stimuli; ours and Czisch's studies used neutral stimuli, thus avoiding the potential preferential processing associated with the participant’s own name. Furthermore, the change in auditory activation in response to a neutral stimulus during sleep was not quantitatively assessed in Portas et al., and thus a decrease in activation in primary auditory cortex could not be ruled out. Put together, our work and previous studies on reduced awareness support a role of high order processing regions as key players in the generation of perceptual awareness.

The results presented here are relevant to the more general issue of the neuronal events that define the threshold of perceptual awareness. Typical experimental paradigms address this fundamental issue by presenting sensory stimuli at the threshold of detection during the awake state [49–53]. However, a major difficulty in such paradigms is that they merely substitute one perceptual stimulus (target) with another (mask, background noise etc.) while subjects are fully conscious under both conditions. A fuller estimation of brain activations linked to conscious awareness should contrast the impact of sensory stimulation during the conscious state, with an identical sensory stimulus when subjects are in the non-conscious state. By taking advantage of the switch between sleep and wakefulness we (as well as previous studies using sensory stimuli during sleep) were able to compare the brain response to identical stimuli under conscious and unconscious states. Our results show that the main effect in which such reduced awareness for language processing is manifested in BOLD-fMRI is in a significant reduction in activity localized to high order language cortex, with a significant but milder reduction in primary auditory activity. In contrast, sub-cortical areas such as the thalamus did not show a significant reduction. Such activity pattern is also reminiscent of attention-like modulations that have been shown to affect primary and high-order audio/linguistic activation [54–56]. However, as noted above, caution should be exercised with regard to the significant activation during sleep, since no behavioral assessment was made as to the level of awareness of participants to the stimuli during the sessions.

The results are in line with a number of previous studies indicating that perceptual awareness is preferentially linked to high rather than low-level stages of the cortical hierarchy. Such results have been consistently documented in the visual system [57–62]. An association between high order processing and sensory awareness has also been suggested for the auditory domain [26]. Thus, the present study extends a line of studies that place a prominent role for high order cortical areas in the emergence of sensory perceptual awareness.

## Materials and Methods

### Participants and Experimental Procedure

Ten right-handed, native Hebrew-speaking healthy volunteers participated in this study (mean±SD age 25±2 years, 4 females); none had neurological or sleeping disorders. Participants gave written informed consent prior to the experimental sessions and all procedures were approved by the Internal Review Board of Shalvata Mental Health Center, Hod-Hasharon, Israel. Participants underwent fMRI scanning on two consecutive nights. In each experimental session, participants fell asleep outside the scanner for a short period of time (~45 minutes) in an attempt to increase the likelihood of falling asleep inside the noisy scanner environment. Afterwards, they were transferred to the MRI scanner, for three hours of scanning or until they felt uncomfortable at each of the experimental nights at around 2:00–5:00 a.m. The participant's head was placed on a foam cushion for stabilization, and MR compatible earphones (MR confon, Magdeburg, Germany) that substantially reduce external noise were placed on the ears. No earplugs were used in order to avoid distortion or uncontrolled attenuation of the auditory stimuli during the night. During the course of the scan an objective physiological measure of either pulse or ECG was acquired in order to construct a sleep-wake indicator (see below). The participants were requested to remain with their eyes closed throughout the experiment, and immediately report if they awoke from a dream in the middle of the scan. No such dream reports were made. The participants were questioned about their experience inside the MRI after each session, and reported their sensations and recollections of periods while they were awake during the scans.

During these 3 hr sessions, participants passively listened to a 9 min sequence of auditory stimuli (see Fig 1). This 9 min sequence was sparsely repeated throughout the night with pseudorandom onset times (each night had different onset times). Each 3 hr session contained 5.7 ± 1.7 auditory sequences (to a total of 11.5 ± 2.2 experiments per participant). Each auditory sequence contained three types of auditory stimuli (duration 4 ± 1.2 s, 9 or 10 repetitions for each type) played at a fixed comfortable non-arousing volume, but sufficiently loud so the stimuli were heard clearly over the scanner noise (volume was adjusted individually for each participant during MRI calibration prior to scanning). Stimuli were interleaved with rest epochs of 11 s or 14 s (Fig 1*b*). The order of the stimuli within a sequence was pseudo randomized, but kept constant in all the sequence repetitions (i.e., each sequence contained the same set of stimuli in the same order). Prior to the beginning of each auditory sequence a 15 s auditory pattern followed by a 9 sec break was presented (pure tunes played at a rapid pace) in order to eliminate the transient response to a novel auditory stimulation, which might artificially increase the response to the first trial.

In a separate session a few days after the night sessions, participants returned for a 9 min auditory localizer experiment during daytime. The localizer session was performed on a separate day in order to make sure the subjects were not drowsy and were fully concentrated.

### Auditory Stimuli

Three categories of auditory stimuli were used with descending semantic content recorded in Hebrew by a female speaker (total of 29 stimuli per experiment) and equalized for volume. One stimuli category was comprehensible sentences containing an ambiguous word (e.g. "*the second went by quickly*, *bypassed the first and won the race*”; similar to [63, 64]; we will henceforth refer to these as “sentences” or “sen”). In the second group of stimuli each word from the original sentences was replaced by a pseudoword with the same number of syllables. The pseudoword sentences had the same prosody and phonemes as the comprehensible sentences, but no intelligible semantic meaning (will be referred to as “pseudoword” or “pse”). In the third category, the comprehensible sentences were temporally divided into 400 ms bins, and the bin order was randomly scrambled using Matlab (MathWorks, Natick, MA) software to create a vocal but non speech-like sound (will be referred to as “scrambled” or “scr”).

### Physiological measurements acquisition

Heart rate or pulse rate was acquired for each participant throughout the scanning sessions. The first three subjects had heart rate measurements, but then because of technical difficulties with the ECG system, and to alleviate the discomfort of participants under the restricting conditions of the MRI scanner, the remaining seven participants were monitored by a pulse electrode attached to their left-hand index finger, as well as an MRI compatible EMG electrode (MP150, Biopac Systems, Inc, Coleta, CA). Additionally, eye movements were recorded via an infrared video camera (SR research eyelink 1000). The pulse was measured by an MRI compatible pressure pad and the signal was conducted outside the scanner room by plastic tubes into a transducer amplifier located in the next room (DA100C, Biopac Systems, Inc, Coleta, CA). The recording commenced with a trigger delivered from the scanner at the beginning of the session. The pulse data was then exported to Matlab using the AcqKnowledge software (Biopac Systems, Inc, Coleta, CA), digitized at 10 kHz and down-sampled to 200 Hz. Next, the signal was sent to an algorithm for local maxima detection (in house software). The time interval between each two consecutive peaks was calculated for either the pulse electrode (seven participants) or the ECG electrode (three participants; heart rate and pulse rate are interchangeable in healthy participants [65]), and the resulting values were converted to units of beats per minute (BPM). The heart rate graph was then square smoothed for visualization purposes. It is important to note in this regard, that our scoring method was based on the longer, slower changes in pulse/heart rates, rather than wave form or transient changes in variability, which might indeed differ between heart rate and pulse measurements.

### Sleep-wake indicator

In order to indicate whether the participants were asleep or awake during each of the auditory sequences, heart rate measures and video monitoring were used [66–69]. For classification to wakefulness or sleep, heart rate data for each night session were plotted (in BPM), and overlaid with grid lines dividing the entire session into 9 min segments, according to the timings of the auditory sequences played to the participants (see e.g., Fig 1*a*). Then three experienced observers independently evaluated the arousal state (awake/asleep/a combination of the two) during each of these 9 min segments (evaluation was performed offline), according to the heart rate graphs. The observers had to decide for each 9 min segment whether the participant was awake or in NREM sleep according to transient elevations or decreases in the heart rate, respectively, taking into account the near history, not just absolute value. Only segments that had both unequivocal state indication (either awake or asleep) for the entire 9 minute segment, and consensus judgment across all three observers were considered for further analysis. All mixed or undetermined state segments were excluded. Additionally, any segments with excessive (>1 mm) or sharp head motion in the middle of the scan resulted in the exclusion of the entire 9 minute segment. Segments with large head movements towards the end of the scan were cropped, so that only the data until the first big motion was used. These measures resulted in some loss of data, but an overall more reliable sleep score.

### Validation procedure of the heart-rate based sleep-wake indicator

Our approach for determining arousal state was validated using a separate data set, collected outside of the scanner, in which conventional EEG based sleep staging was combined with heart rate measurements. Thus, an independent dataset of 20 participants' full night EEG traces together with their ECG (heart rate) measurements outside the MRI were taken as test data (data taken from [70]). Standard EEG sleep staging was performed manually by an experienced scorer (AA), and heart rate data was extracted from the ECG channel. The heart rate graphs were overlaid with grid lines emulating borders between 9 min segments (in a similar manner to those drawn in our experimental data). Each session lasted for 313.2±8 min resulting in 34.8±0.9 segments per participant. Then, the three experienced observers independently classified the segments to awake/sleep/a combination of the two, according to heart rate alone. The observers were blind to each other's classifications as well as to the EEG manual scoring results. The mean rate of consent across the three observers was 66±3%, out of which 40±1% were indicated as non-mixed sleep or wakefulness (35% sleep and 5% wakefulness). S1*a* Fig shows an example of the manual sleep staging together with the heart rate data (in BPM). Only the segments that received consensus scoring across the three observers as either sleep or wakefulness according to heart rate were included in the validation assessment. Each of these segments was given a score of either stage1/stage2/slow wave sleep (SWS)/wakefulness/REM according to the manual EEG-based scoring. Because more detailed sleep staging was possible in the manual scoring, some of the segments were assigned a combination of several stages. The segments were then pooled across participants, and the proportion of each EEG-based stage was calculated separately for segments classified as awake and for segments classified as sleep according to pulse rate (S1*b* Fig). As can be seen, the heart rate scoring was 99.6% successful at identifying NREM sleep states correctly (however it also captured some mixed segments, and one segment that was classified as REM) and 100% successful at identifying awake/REM state segments (however some mixed segments were also included).

### Differentiating wakefulness from REM sleep

As shown in S1b Fig, using the heart rate measure can create confusion between REM and wakefulness (as with EEG measures), as both are characterized by elevation in heart rate. Searching for REM periods in the segments judged to be awake was conducted, in all ten participants in the experiment by searching for reduced EMG amplitude traces or the occurrence of saccadic eye movements [71]. However, no incidents of REM sleep were detected during the MRI scans, which was confirmed also by post-scan interviews with each participant.

### Permutation test for sleep-wake labeling

To further validate our labeling of auditory sequences as either wakefulness or sleep, we performed a permutation test as follows: for auditory cortex ROI, the beta values of each participant along with their labels (sleep/awake) were considered. The labels of the beta values were then shuffled within the runs of each participant, for a total of 10,000 iterations. For each such iteration, the mean (shuffled) awake beta and mean (shuffled) sleep betas were calculated for each participant (resulting in one single beta value per subject for sleep and one for awake), a repeated measures ANOVA was performed, and the F statistic for the state factor (awake/sleep) was recorded. The significance level of the original unshuffled F value was then calculated as follows: B = number of F values that were greater than or equal to the unshuffled F value. M = number of iterations. $p\;=\;\frac{B+1}{M+1}.$

### Imaging Setup and fMRI Data Analysis

Images were acquired on a 3 Tesla scanner (Tim Trio, Siemens), equipped with a receive-only 12 –channels head matrix coil. Functional T2*-weighted images were acquired with a gradient echo EPI sequence with a repetition time (TR) of 3000 ms, an echo time (TE) of 30 ms, a 90° flip angle, a resolution of 3x3x3 mm, acquiring 46 transverse slices tilted to the AC-PC plane, covering the whole brain without gaps. Three dimensional gradient echo T1-weighted anatomical images were acquired to facilitate the incorporation of the functional data into the 3D Talairach space. The anatomical images were acquired with a resolution of 1x1x1 mm using the 3D-MPRAGE sequence with TR/TE/inversion time (TI)/flip angle of 2300ms/2.98 ms/900 ms/9°.

fMRI data were analyzed with the "Brain-voyager" software package (Brain Innovation, Masstricht, Netherlands) and with custom software written in Matlab (Mathworks, Natick, MA). The first two images of each functional scan were discarded, and the remaining functional images were then incorporated into the 3D data sets through trilinear interpolation. Preprocessing of functional scans included 3D motion correction, filtering out of low frequencies up to 3 cycles per scan (slow drift), and spatial smoothing using Gaussian filter of 8 mm. Segments or parts of segments showing movement artifacts larger than 1 mm or sharp head movements were excluded from the analysis. The cortical surface in a Talairach coordinate system was reconstructed for each participant from the 3D- gradient echo anatomical scan. Statistical analytic mapping was based on the general linear model (GLM), with a regressor for each auditory stimulus category corresponding to its specific protocol. A canonical hemodynamic lag was assumed for each participant. The analysis was performed independently for the time-course of each individual voxel, and after computing the beta coefficients for the regressor, a Student’s t test was performed. Multi-subject analysis was based on a random-effect GLM, and the multi-participant functional maps were then projected on an inflated Talairach normalized brain. Significance levels were calculated, with correction for multiple comparisons performed by taking into account the minimum cluster size and the probability threshold of a false detection of any given cluster. This was accomplished by a Monte Carlo simulation (AlphaSim by B. Douglas Ward), using individual voxel probability threshold; the probability of a false positive detection per image was determined from the frequency count of cluster sizes within the entire brain surface.

### Definition and analysis of Regions of Interest (ROIs)

ROIs were defined for each participant individually according to his independent auditory localizer session. Each ROI included voxels above threshold (FDR < 0.05) in the relevant anatomical locations (see results section for details). The thalamic ROI, (size 37 ± 12 EPI voxels) was defined as the voxels above threshold when contrasting all auditory stimulus types versus rest (Fig 3). Primary auditory (size 126 ± 16 EPI voxels) ROI was defined as voxels that were activated above threshold for scrambled sentences versus rest (Fig 2*a*).

Wernicke ROI (size 627 ± 260 EPI voxels), and Broca ROI (size 161 ± 78 EPI voxels) were defined by considering the voxels above the threshold when contrasting comprehensible (high level of semantic content) and scrambled (no semantic content) sentences (Fig 2*b*). ROI analyses were carried out by averaging across voxels to compute a single response time course for each ROI in each individual auditory sequence. The average percentage of event-related BOLD signal change was calculated per condition for each participant (considering time points between -2 and 5 TRs from stimulus onset; file based baseline defined as the 2 TRs prior to stimulus onset), and was then averaged across participants. In addition, mean beta values, derived from a general linear model (GLM), were calculated in each ROI for each condition (sleep/wake) and each participant. The GLM was calculated for each 9 min epoch independently, then the resulting beta values for each run were averaged such that each participant obtained one mean beta value for wakefulness runs and another mean beta value for the sleep runs (irrespective of the number of runs obtained for each participant). All multi-subject analyses were then carried out on this single mean beta value per subject/condition. These mean beta values were then taken for a repeated measures ANOVA with two factors (state factor: sleep/awake, condition factor: comprehensible sentence/pseudoword/scrambled) was performed on the beta values using STATISTICA software (Statsoft inc., Tulsa, OK), followed by an ad-hoc Tukey test. Additionally, one-sample t tests were performed to determine which ROIs showed significant positive responses across participants in each condition, and corrected for multiple comparisons using the Bonferroni method.

Next, an index of selectivity for comprehensible sentences was calculated by subtracting the beta values of the scrambled sentences category from the comprehensible sentences category (sen—scr) in each of the four ROIs (Fig 5*a*). Finally, a second index assessing the effect of sleep on the response to comprehensible sentences was calculated for each ROI as the betas for comprehensible sentences category in: (sleep—awake)/awake (Fig 5*b*). For this analysis the median beta values rather than the mean values were used, as there was one subject who was a significant outlier. A Mann Whiteny u test was used to compare the different ROIs, followed by Bonferroni corrections for multiple comparisons.

## Supporting Information

S1 Fig Comparison between heart rate-based staging and EEG-based staging in an independent test data set outside the MRI. (*a*) An example trace of a test participant showing manual scoring of sleep stage with EEG, performed according to standard criteria (Rechtschaffen and Kales, 1968) during the night, together with heart rate calculated as beats per minute according to ECG electrode data. The Y axis denotes the momentary sleep stage (EEG-based score) or the normalized heart rate. Grid divides the x axis into equal segments of 9 min each. Rectangles signify segments that received consensus scoring across the three observers as either sleep or wakefulness according to heart rate alone (gray = sleep; yellow = awake). (*b*) Distribution of EEG-based stages in segments classified according to heart rate as NREM sleep or awake/REM in 20 test subjects (data taken from Arzi et al., 2010). HR = heart rate, S1 = stage1, S2 = stage2, SWS = slow wave sleep.
(TIF)

S2 Fig Distribution of undisturbed sleep epoch length for all participants, across both nights. Sleep epochs are scored in nine-minute segments, as per our scoring method (see methods).
(TIF)

S3 Fig Right hemisphere ROIs analysis of all three types of auditory stimuli in wakefulness and sleep during night sessions. For details, see Fig 4 in main text.
(TIF)

S1 Table Confusion matrix showing the classification of the independent test set data according to both the heart-rate based staging, and the EEG-based staging. (DOCX)
