# Supplementary figures and images for: Diminished Auditory Responses during NREM Sleep Correlate with the Hierarchy of Language Processing
Source: PLoS One. 2016 Jun 16;11(6):e0157143. doi: 10.1371/journal.pone.0157143 (PMC4911044; doi:10.1371/journal.pone.0157143)

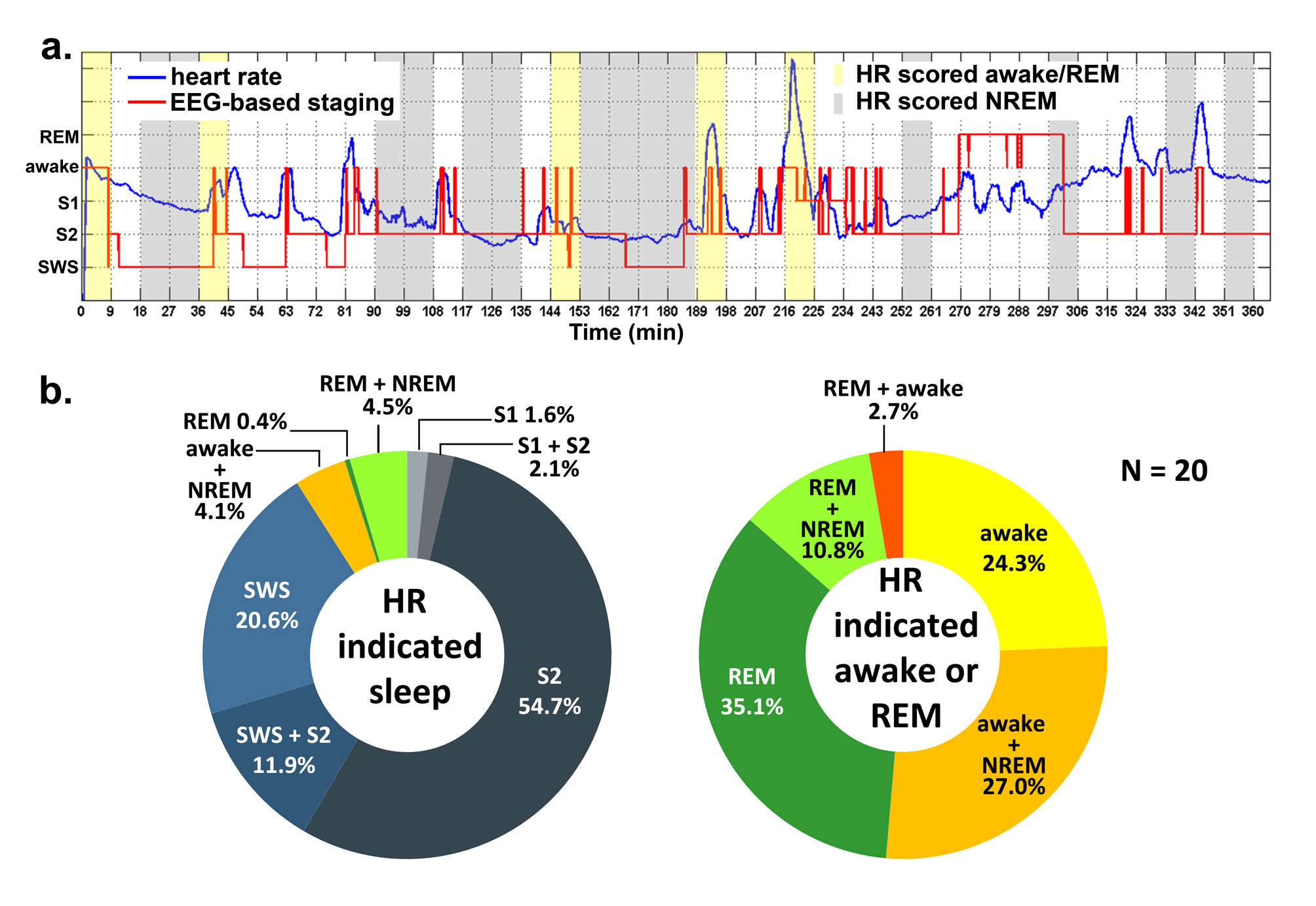

Supplement: S1 Fig — (a) An example trace of a test participant showing manual scoring of sleep stage with EEG, performed according to standard criteria (Rechtschaffen and Kales, 1968) during the night, together with heart rate calculated as beats per minute according to ECG electrode data. The Y axis denotes the momentary sleep stage (EEG-based score) or the normalized heart rate. Grid divides the x axis into equal segments of 9 min each. Rectangles signify segments that received consensus scoring across the three observers as either sleep or wakefulness according to heart rate alone (gray = sleep; yellow = awake). (b) Distribution of EEG-based stages in segments classified according to heart rate as NREM sleep or awake/REM in 20 test subjects (data taken from Arzi et al., 2010). HR = heart rate, S1 = stage1, S2 = stage2, SWS = slow wave sleep. (TIF) [file pone.0157143.s001.tif]

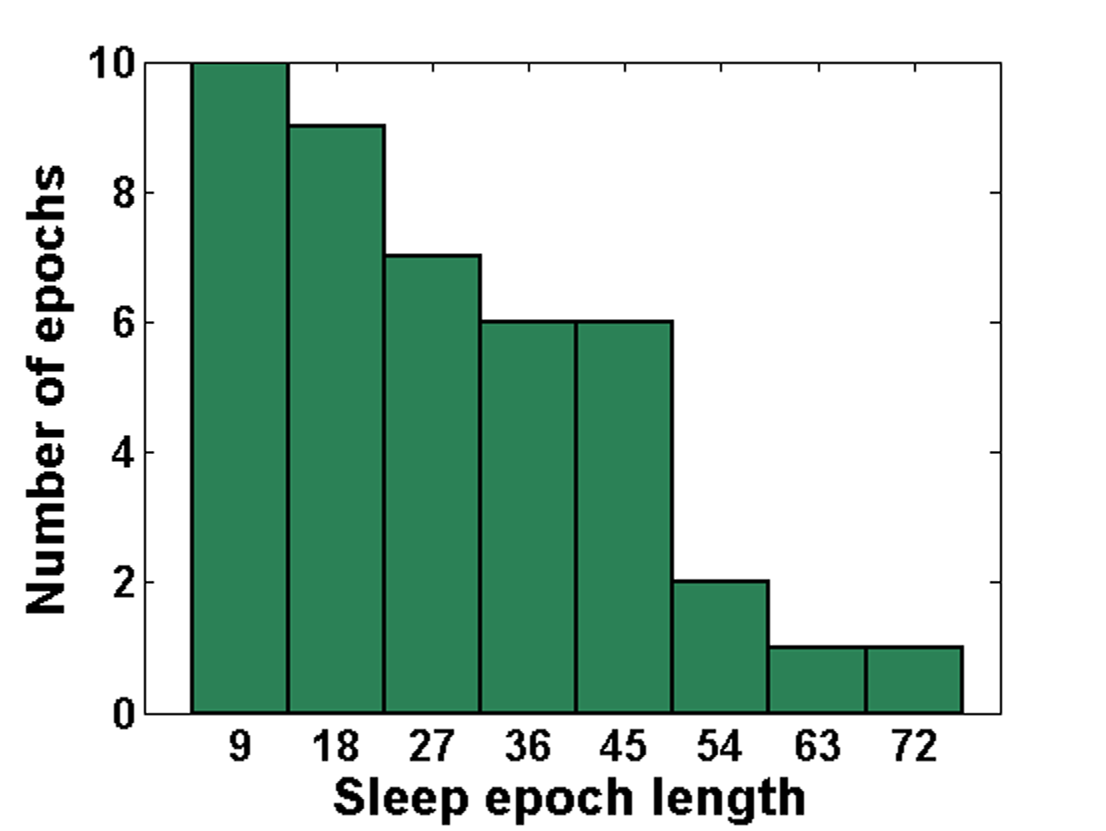

Supplement: S2 Fig — Sleep epochs are scored in nine-minute segments, as per our scoring method (see methods). (TIF) [file pone.0157143.s002.tif]

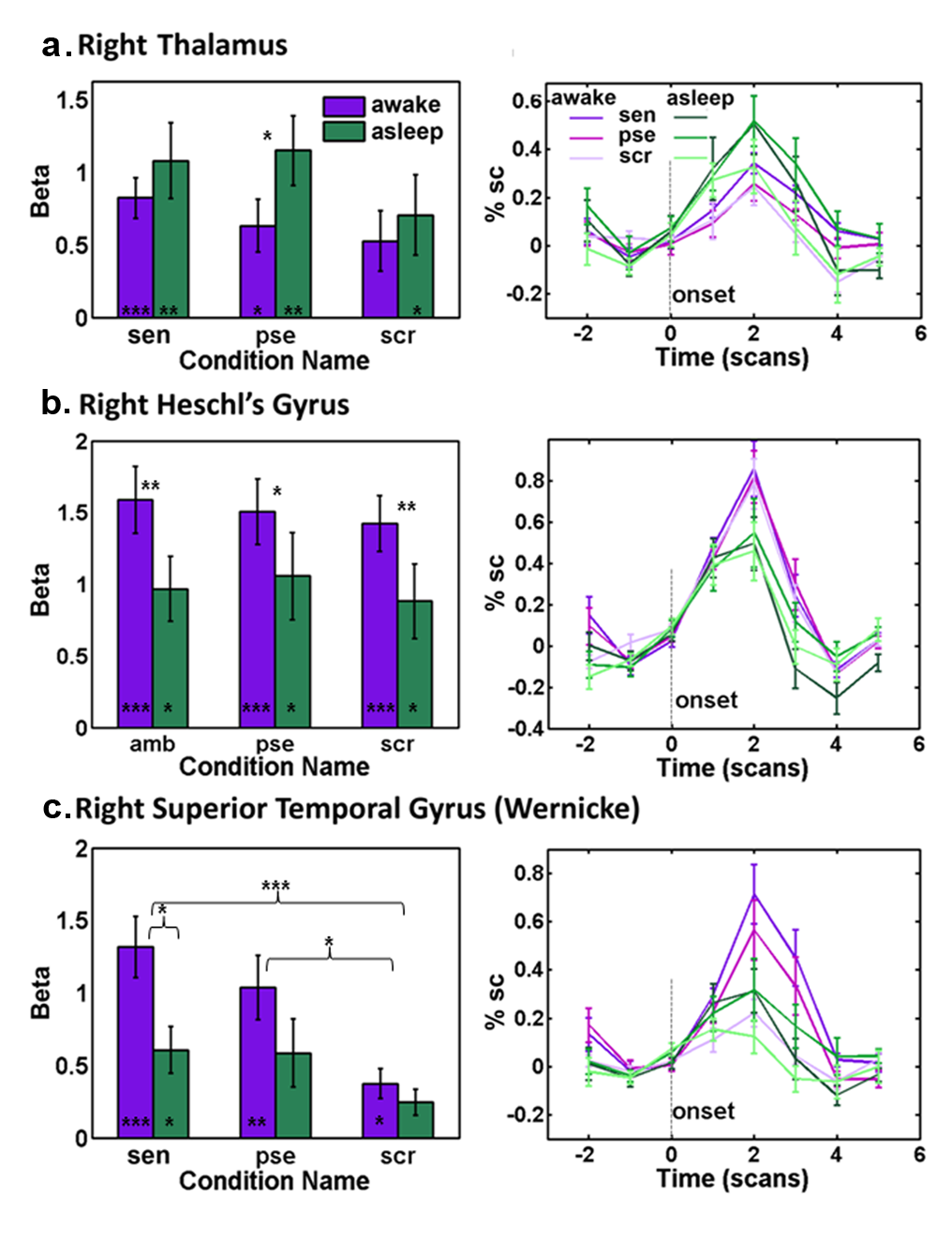

Supplement: S3 Fig — For details, see Fig 4 in main text. (TIF) [file pone.0157143.s003.tif]
