# Supplementary material for: Diminished Auditory Responses during NREM Sleep Correlate with the Hierarchy of Language Processing
Source: PLoS One. 2016 Jun 16;11(6):e0157143. doi: 10.1371/journal.pone.0157143 (PMC4911044; doi:10.1371/journal.pone.0157143)
Supplement: S1 Table — (DOCX) [file pone.0157143.s004.docx]

**Table S1.** Confusion matrix showing the classification of the independent test set data according to both the heart-rate based staging, and the EEG-based staging.

| **Manual EEG-based staging** | **HR indicated sleep** | **HR indicated awake/REM** |
| --- | --- | --- |
| awake | 0 | 9 |
| awake + REM | 0 | 1 |
| awake + REM + stage2 | 0 | 1 |
| awake + stage1 | 0 | 3 |
| awake + stage2 | 8 | 5 |
| awake + SWS | 2 | 1 |
| REM | 1 | 13 |
| REM + stage2 | 10 | 4 |
| stage1 | 4 | 0 |
| stage1 + REM | 1 | 0 |
| stage1 + stage2 | 5 | 0 |
| stage2 | 132 | 0 |
| stage2 + SWS | 29 | 0 |
| SWS | 50 | 0 |
| **total** | **242** | **37** |

* HR=heart rate
